# Evolution of retiform hemangioendothelioma into angiosarcoma

**DOI:** 10.1016/j.jdcr.2025.10.015

**Published:** 2025-10-24

**Authors:** Nicole J. Hardy, Samantha Mallari, Donna Aiudi, Ga Hie Nam, Wahila Alam

**Affiliations:** a University of Connecticut School of Medicine, Farmington, Connecticut; b Department of Dermatology, University of Connecticut School of Medicine, Farmington, Connecticut; c Department of Pathology and Laboratory Medicine, University of Connecticut School of Medicine, Farmington, Connecticut; d Department of Geriatrics, University of Connecticut School of Medicine, Farmington, Connecticut

**Keywords:** cutaneous, metastatic, oncology, Retiform hemangioendothelioma, sarcoma

## Introduction

Retiform hemangioendothelioma (RHE), composite hemangioendothelioma (CHE), and angiosarcoma (AS) are rare vascular tumors that commonly involve the skin and subcutaneous tissues, but they differ markedly in biology and clinical behavior.^1^ RHE and CHE are considered to have intermediate malignant potential with frequent local recurrence but rare distant metastasis.^2^ On microscopic examination of RHE, the vessels resemble the rete testis with a network pattern, and the vascular spaces are lined by protuberant endothelial cells (hobnail nuclei). Immunohistochemical staining is generally positive for vascular markers such as CD31, CD34, and ERG. On the other hand, the histology of CHE will show a mixture of hemangioendothelioma subtypes, and AS will show anastomosing vessels, high mitotic activity, and pleomorphic cells with prominent nucleoli.^3^ While AS-like areas are found in approximately half of CHE cases, it is exceedingly rare for it transform into a high-grade AS.^4^

## Case report

**Fig 1:**
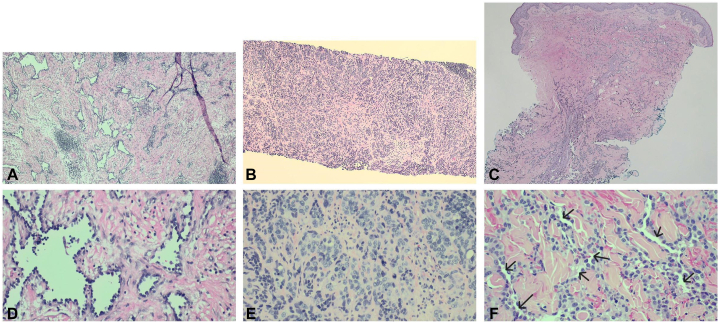
Histology of the lesion at 3 different time points demonstrating evolution from retiform hemangioendothelioma to composite hemangioendothelioma to angiosarcoma. **A,** Retiform hemangioendothelioma on excision specimen of the right shoulder (H&E stained sections, ×40 magnification) showing elongated branching vascular channels within edematous and collagenous stroma. **B,** Composite hemangioendothelioma on core needle biopsy specimen of the right neck (H&E stained sections, ×40 magnification) showing infiltrative solid nests of hypercellular tumor cells. **C,** Angiosarcoma on skin punch biopsy specimen of the left chest (H&E stained sections, ×20 magnification) showing ill-defined tumor cells with anastomosing vessels/vasoformation. **D,** Retiform hemangioendothelioma on excision specimen of the right shoulder (H&E stained sections, ×200 magnification) showing hobnail endothelial cells with protuberant hyperchromatic nuclei within endematous and colagenous stroma without multilayering. **E,** Composite hemangioendothelioma on core needle biopsy specimen of the right neck (H&E stained sections, ×400 magnification) showing increased cytologic atypia and frequent mitosis. **F,** Angiosarcoma on skin punch biopsy specimen of the left chest (H&E stained sections, ×200 magnification) showing apparent anastomosing vascular channels *(arrows)* lined by cells with marked large vesicular nuclei, prominent nucleoli, frequent mitosis, and multilayering.

A previously healthy 34-year-old man who worked as a civil engineer noticed a small red spot on his right shoulder a few millimeters in diameter. The lesion was excised at an outside clinic, but the pathology was unavailable. Three years later, a pedunculated, mobile, painless mass recurred in the same location. This lesion was excised. Pathology showed hobnail endothelial cells with protuberant hyperchromatic nuclei and elongated branching vascular channels, consistent with RHE with focal positive margins (Fig 1, *A* and *D*). The specimen stained positive for YAP1, ERG, and CD31, and equivocal for MAML2.

**Fig 2:**
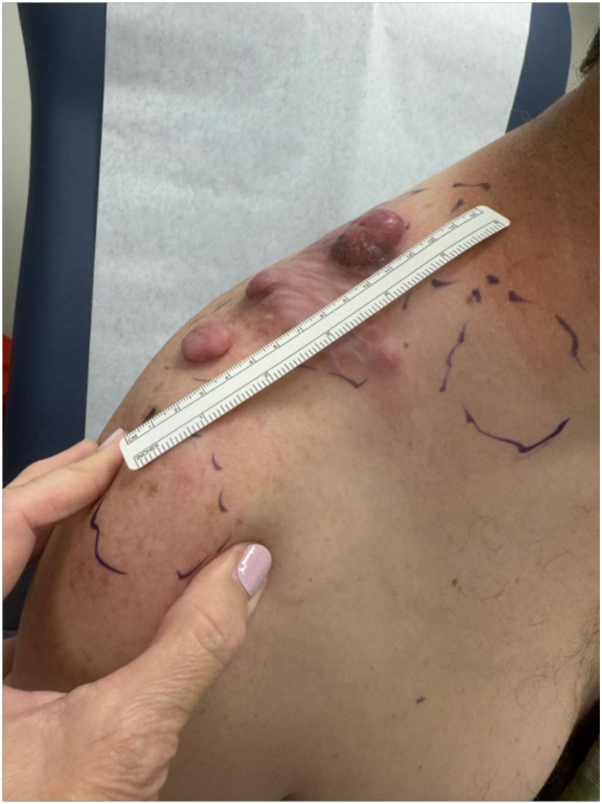
Multiple dome-shaped nodules on the posterior right shoulder, ranging from 1 to over 3 cm in diameter. The lesions are *pink* to violaceous, firm, and shiny, with stretched overlying skin. The photo was taken 4 months before his death.

**Fig 3:**
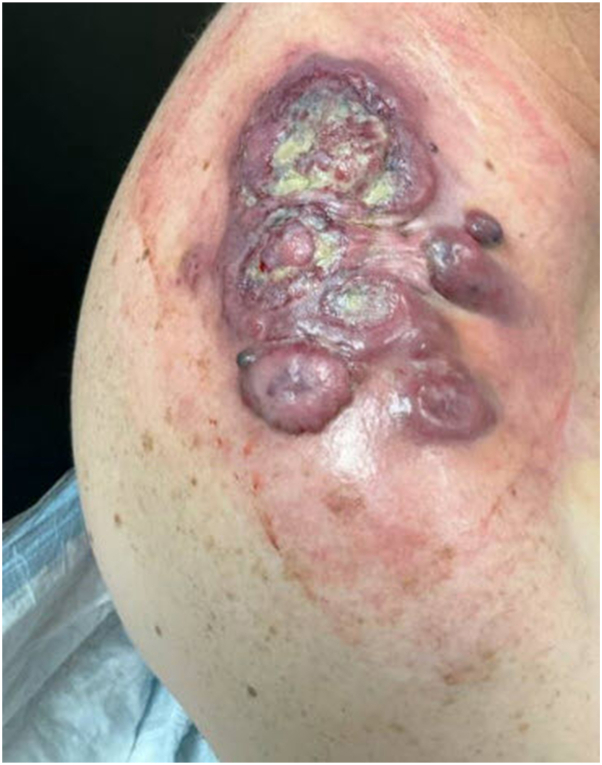
A 9.3 cm × 8.5 cm × 6.4 cm lesion on the right shoulder of the patient demonstrating violaceous-to-dusky, dome-shaped dermal/subcutaneous nodules with *yellow-green* purulent material and ulceration. This photo was taken 1 month before his death.

**Fig 4:**
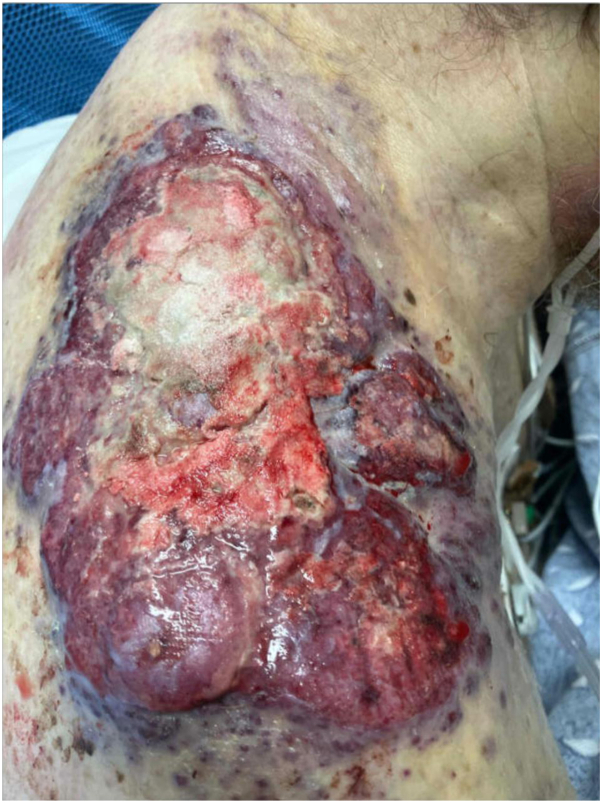
Large ulcerated vascular tumor in the right shoulder/neck region demonstrating a violaceous-to-erythematous, exophytic mass with irregular borders. The lesion shows extensive ulceration with hemorrhagic and necrotic areas, friable tissue, and fibrinous exudate. The heterogeneous surface, rapid progression, and infiltrative appearance are features highly suggestive of cutaneous angiosarcoma. This image was taken 2 days before he died.

The patient was lost to follow-up for 3 years but later sought medical attention when he found a hard lump in his right neck region, near where the first lesion appeared. Over the next year, this lump increased in size, and a second lesion appeared nearby over his right shoulder (Fig 2, Fig 3, Fig 4). The biopsy the neck lesion demonstrated infiltrative solid nests with both epithelioid and focally retiform architectures and increased mitotic figures (8 mitosis per 10 HPF), meeting WHO diagnostic criteria for CHE, which requires the presence of at least 2 morphologically distinct vascular tumor elements (Fig 1, *B* and *E*). This interpretation was further supported by the patient’s prior history of RHE. The diagnosis of CHE was independently confirmed by soft tissue expert pathologists at 3 separate institutions (Memorial Sloan Kettering Cancer Center, Brigham and Women’s Hospital, and Mayo Clinic).

As part of the workup, the patient received a pan CT scan with contrast, which demonstrated multiple enhancing cutaneous and subcutaneous lesions in the right posterior and lateral neck. His PET scan demonstrated multiple FDG-avid lymph nodes and skeletal lesions and small bilateral pleural effusions, suspicious for malignancy and metastatic disease. A month later, he had multiple admissions for pleural effusions. A punch biopsy of his left chest (Fig 1, *C* and *F*) showed a CHE with increased cytologic atypia, consistent with evolution to AS. In contrast to the previous biopsies, the diagnosis of AS was based on the presence of anastomosing vascular channels with a higher degree of cytologic atypia.

During admission, he was initiated on pazopanib, a tyrosine kinase inhibitor used in the treatment of soft tissue sarcomas. His hospital course was prolonged due to cancer-related pain and high-output from his bilateral pleural effusion. The patient ultimately passed away from septic shock and respiratory failure approximately 6 years after the appearance of his first tumor.

## Discussion

This is a case report of a patient with RHE evolving into CHE and AS with distant metastases. Distant metastasis in RHE is rare, and transformation into AS is even less common with only 1 reported case to date that has convincingly demonstrated evolution from CHE to AS.^5^ Our case similarly demonstrates changes in histomorphology and degree of cytologic atypia that support the evolution into AS. Importantly, the diagnostic distinction of the neck lesion as CHE rather than AS is supported by both WHO criteria and multi-institutional expert pathology review, underscoring that the diagnosis was not based on a single feature but on a constellation of morphologic elements and expert consensus. The clinical course of the patient further supports the evolution to AS; the 5-year survival rate is just 26.4% across all stages and <10% for Stage 4.^6^ Therefore, it is unlikely that the patient had AS from his initial presentation. This case presents evidence of transformation from RHE into CHE and then into AS. It highlights that while rare, transformation can occur, and that appropriate patient counseling is imperative to ensure adequate surveillance.

## Conflicts of interest

None disclosed.
